# Identification and characterization of two trypanosome TFIIS proteins exhibiting particular domain architectures and differential nuclear localizations

**DOI:** 10.1111/j.1365-2958.2008.06348.x

**Published:** 2008-07-15

**Authors:** Pierrick Uzureau, Jan-Peter Daniels, David Walgraffe, Bill Wickstead, Etienne Pays, Keith Gull, Luc Vanhamme

**Affiliations:** 1 Laboratoire de Parasitologie MoléculaireULB IBMM, rue des Pr Jeneer et Brachet 12, B-6041 Gosselies, Belgium; 2 Sir William Dunn School of Pathology, University of OxfordSouth Parks Road, OX1 3RE Oxford, UK

## Abstract

Nuclear transcription of *Trypanosoma brucei* displays unusual features. Most protein-coding genes are organized in large directional gene clusters, which are transcribed polycistronically by RNA polymerase II (pol II) with subsequent processing to generate mature mRNA. Here, we describe the identification and characterization of two trypanosome homologues of transcription elongation factor TFIIS (TbTFIIS1 and TbTFIIS2-1). TFIIS has been shown to aid transcription elongation by relieving arrested pol II. Our phylogenetic analysis demonstrated the existence of four independent TFIIS expansions across eukaryotes. While TbTFIIS1 contains only the canonical domains II and III, the N-terminus of TbTFIIS2-1 contains a PWWP domain and a domain I. TbTFIIS1 and TbTFIIS2-1 are expressed in procyclic and bloodstream form cells and localize to the nucleus in similar, but distinct, punctate patterns throughout the cell cycle. Neither TFIIS protein was enriched in the major pol II sites of spliced-leader RNA transcription. Single RNA interference (RNAi)-mediated knock-down and knockout showed that neither protein is essential. Double knock-down, however, impaired growth. Repetitive failure to generate a double knockout of TbTFIIS1 and TbTFIIS2-1 strongly suggests synthetical lethality and thus an essential function shared by the two proteins in trypanosome growth.

## Introduction

Trypanosomes are a group of flagellated protozoa including parasitic species such as *Leishmania major*, *Trypanosoma cruzi* and *T. brucei*, which are responsible for major diseases. They are transmitted to their mammalian host during a blood meal of an insect vector. Nuclear transcription of trypanosomes displays unusual features compared with other well-studied eukaryotic systems (see Campbell *et al*., 2003; Palenchar and Bellofatto, 2006).

RNA polymerase II (pol II)-transcribed protein-coding genes are organized in large directional gene clusters (DGCs) which can span more than one megabase and are separated by strand-switch regions (SSRs) (Berriman *et al*., 2005; El-Sayed *et al*., 2005; Ivens *et al*., 2005). In *T. brucei*, the average cluster is 230 kb long and contains 70 genes (Hertz-Fowler *et al*., 2007). The pol II transcripts from the DGCs are polycistronic and are co-transcriptionally processed to monocistronic mRNAs by *trans*-splicing a short, capped spliced-leader (SL) exon to the 5′ UTRs and polyadenylation of the 3′ UTRs. It is not understood whether pol II initiates and terminates only in SSRs or whether it can start transcription promiscuously throughout the DGC (see Campbell *et al*., 2003). The former would require a high processivity of pol II in trypanosomes. In other eukaryotic systems studied so far, this property of pol II has been found to depend on the recruitment of transcription elongation factors (see Arndt and Kane, 2003).

In the bloodstream parasite *T. brucei*, RNA polymerase I (pol I) not only transcribes the 45S rRNA precursor, but also genes located in the polycistronic variable surface glycoprotein (VSG) antigen expression sites (ESs) (see Pays *et al*., 2004). There are multiple transcription-competent ESs, located adjacent to telomeres. The escape of *T. brucei* from the immune response of its mammalian host by antigenic variation requires the mono-allelic expression of only a single ES at any one time. While arrest of pol I on one of the short and numerous rDNA repeats will probably not be harmful to a cell, an RNA polymerase arrested on the single active ES, which can be more than 100 kb in size, would compromise VSG expression. Interestingly, transcription does initiate on the inactive ESs but the elongation of the transcript to the downstream *VSG* gene does not take place (Vanhamme *et al*., 2000).

Analysis of trypanosome genome sequences has revealed that many of the basal set of eukaryotic pol II transcription initiation factors are either absent or too divergent to be identified by standard bioinformatic queries (Ivens *et al*., 2005). While several studies have addressed the composition of the nuclear RNA polymerase complexes in *T. brucei* (Schimanski *et al*., 2003; 2005; Walgraffe *et al*., 2005; Devaux *et al*., 2006; 2007; Nguyen *et al*., 2006; 2007), information on transcription factors associated with them is very limited. Among the transcription factors that have been studied in trypanosomes, only homologues of TFIIH subunits, which are important for transcription-coupled repair, and the chromatin remodelling factor ISWI might be involved in transcription elongation (Hughes *et al*., 2007; Lecordier *et al*., 2007; Lee *et al*., 2007).

In eukaryotic transcriptional systems, TFIIS is one of the best-characterized transcription elongation factors (see Shilatifard *et al*., 2003; Sims *et al*., 2004). When the elongation of pol II is impaired, the enzyme pauses. Subsequent backtracking of pol II leads to a displacement of the 3′ hydroxy end of the nascent transcript from the active site of the enzyme and can thus result in an arrested state, which can only be resolved by an external factor (see Fish and Kane, 2002). TFIIS can relieve the arrest of pol II and thus provides the transcribing complex with a new opportunity to overcome the obstacle on the template. In addition, two recent reports demonstrated the implication of TFIIS in pol II pre-initiation complex formation, independent of its function as elongation factor (Guglielmi *et al*., 2007; Kim *et al*., 2007).

The understanding of the molecular mechanism of TFIIS function has been dependent on the study of its domain architecture and structure (Awrey *et al*., 1998; Olmsted *et al*., 1998; Booth *et al*., 2000). The canonical TFIIS is composed of three domains. The most N-terminal one, termed domain I, is dispensable for TFIIS function in *Saccharomyces cerevisiae* (Nakanishi *et al*., 1995). In humans, however, it has been shown to interact with the pol II complex (Pan *et al*., 1997) and its LW motif is involved in nuclear targeting (Ling *et al*., 2006). The central domain II and the C-terminal domain III of TFIIS are essential for the function of the protein (Nakanishi *et al*., 1995). Structural analyses of TFIIS on arrested pol II have shown that domain II binds to the jaw of the polymerase, the interdomain linker extends into the funnel and the four-cystein zinc ribbon fold of domain III attaches to the pore (Kettenberger *et al*., 2003; 2004). A thin beta-hairpin extrudes from the zinc ribbon fold into the pore to the active site of pol II. A conserved pair of acidic amino acid residues (aspartate and glutamate) complements the active site to allow the cleavage of the nascent transcript. This provides a new 3′ hydroxy for pol II to continue transcription.

Deletion of TFIIS in *S. cerevisiae* does not impair cell growth under optimal conditions. However, a phenotype was revealed when the cells were propagated in the presence of 6-azauracil (6-AU) and mycophenolic acid (MPA) (Nakanishi *et al*., 1992; Wery *et al*., 2004). These drugs are thought to lower the availability of nucleotides in the cell, which causes transcriptional pauses and thus an increase in the dependency on transcription elongation factors (Exinger and Lacroute, 1992). While yeasts harbour only one TFIIS gene, three paralogues have been detected in vertebrate genomes (Labhart and Morgan, 1998), which are most divergent in their domain I to domain II interdomain linker. The three proteins have been shown to display some tissue specificity of expression, possibly indicating functional divergence (see Wind and Reines, 2000). Deletion of one paralogue, *TCEA1*, is lethal during embryonic development in mice due to failure of definitive haematopoiesis (Ito *et al*., 2006).

Here, we report the bioinformatic identification and subsequent characterization of two TFIIS proteins in *T. brucei*, TbTFIIS1 and TbTFIIS2-1, with different domain architectures. We analysed their expression and subcellular localization in procyclic and bloodstream form cells. Also, we evaluated the requirement of these factors for cell growth under normal conditions and under transcription-affecting stresses.

## Results

### Identification of two domain III-containing TFIIS homologues in *T. brucei*

**Fig. 1:**
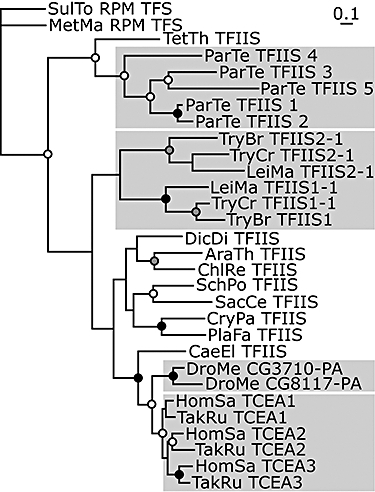
Phylogeny of eukaryotic TFIIS. A Bayesian TFIIS phylogeny encompassing 30 non-redundant sequences from 16 diverse eukaryotic and two archaeal organisms with complete or near-complete genome sequence (see *Experimental procedures* for details). The tree is arbitrarily rooted using SulTo_RPM_TFS (NP_378260) and MetMa_RPM_TFS (NP_633422) as out-group. Prefixes are as follows: AraTh, *Arabidopsis thaliana*; CaeEl, *Caenorhabditis elegans*; CryPa, *Cryptosporidium parvum*; ChlRe, *Chlamydomonas reinhardtii*; DicDi, *Dictyostelium discoideum*; DroMe, *Drosophila melanogaster*; HomSa, *Homo sapiens*; LeiMa, *Leishmania major*; MetMa, *Methanosarcina mazei*; ParTe, *Paramecium tetraurelia*; PlaFa, *Plasmodium falciparum*; SacCe, *Saccharomyces cerevisiae*; SchPo, *Schizosaccharomyces pombe*; SulTo, *Sulfolobus tokodaii*; TakRu, *Takifugu rubripes*; TryBr, *Trypanosoma brucei*; and TetTh, *Tetrahymena thermophila*. For accession numbers, see Table S2. Support for the inferred topology from Bayesian (B), maximum-likelihood (ML) and neighbor-joining (NJ) bootstrap replicates is indicated by white (B and ML ≥ 0.50), grey (all three ≥ 0.75) and black (all three ≥ 0.95) node circles respectively (see *Experimental procedures* for details). Grey boxes indicate the four independent TFIIS multiplication events resulting in multiple TFIIS proteins in *P. tetraurelia*, trypanosomatids, *D. melanogaster* and vertebrates.

To identify TFIIS factors in the trypanosome genomes, we conducted an iterative profile-hidden Markov model search for TFIIS homologues from a seed of well-characterized TFIIS sequences (see *Experimental procedures* for details). The model was then used to search the predicted proteomes of two archaeal and 16 eukaryotic, evolutionary diverse organisms. Eukaryotic proteins that did not pass our criteria for sufficient similarity to both domains II and III were excluded from the output. This resulted in a data set of 28 eukaryotic and two archaeal proteins (Fig. 1). Each trypanosome predicted proteome investigated contributed two orthologues to this list. In *T. brucei* and *L. major*, we subsequently annotated these as TbTFIIS1 (Tb11.02.2600), LmTFIIS1-1 (LmjF24.0210), TbTFIIS2-1 (Tb927.2.3580) and LmTFIIS2-1 (LmjF33.2810). Additionally, in *T. cruzi* and *L. major*, a protein similar to TcTFIIS1-1 and LmTFIIS1-1 was identified and termed TFIIS1-2 (LmjF24.0200). Interestingly, the chromosomal location of its gene is directly adjacent to the respective *TFIIS1-1* gene. *TFIIS1-2* appears to be absent from the *T. brucei* genome. Similarly, TFIIS2-1 has a paralogue in each of the three trypanosome-predicted proteomes analysed, which we termed TFIIS2-2. In all cases, its gene is located directly next to the respective *TFIIS2-1* gene in the genome (Tb927.2.3480; LmjF33.2820) (data not shown). However, as amino acid residues in domain III known to be critical for TFIIS function are mutated (in TcTFIIS2-2 and LmTFIIS2-2) or domain III is even absent (in TbTFIIS2-2), these proteins cannot function as canonical TFIIS. LmTFIIS1-1, LmTFIIS1-2 and LmTFIIS2-1 and its orthologues in *T. brucei* and *T. cruzi* are among four proteins previously suggested as being putative TFIIS factors (Ivens *et al*., 2005). The fourth protein (Tb11.01.2190; LmjF36.4145) has meanwhile been annotated as RPA12, a subunit of pol I homologous to TFIIS (Kelly *et al*., 2005; Walgraffe *et al*., 2005).

**Fig. 2:**
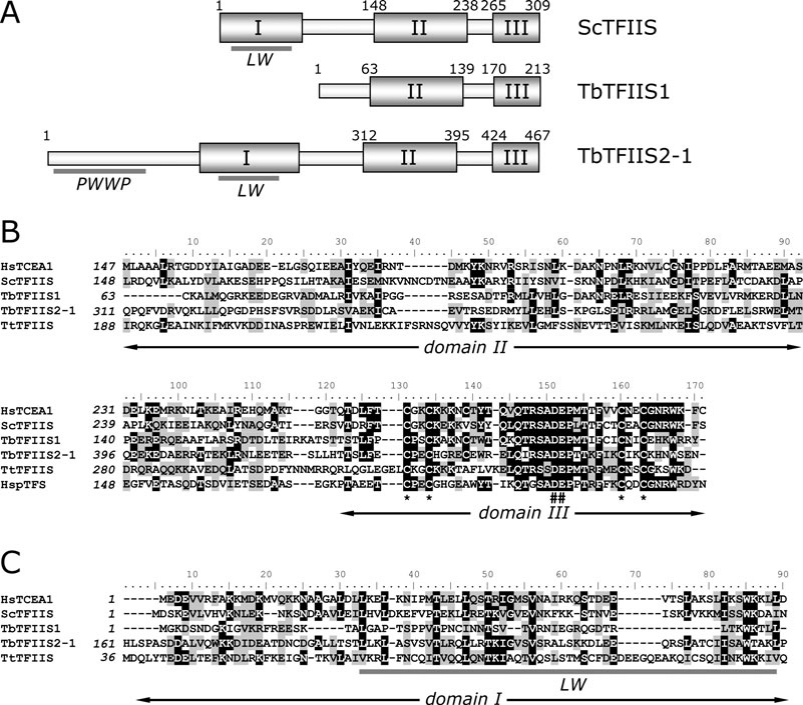
Identification of two domain III-containing TFIIS proteins in the *T. brucei* genome. A. Schematic of the ScTFIIS, TbTFIIS1 and TbTFIIS2-1 proteins. The boundaries of three TFIIS domains are indicated as boxes and numbered when known. The PWWP domains and LW motifs are indicated as thick grey lines. B and C. Multiple sequence alignments of TFIIS proteins. Analyses were performed using the following sequences and protein database accession numbers: HsTCEA1 (AAH72460.1), ScTFIIS (P07273), TtTFIIS (EAR84422.2), HspTFS (AAG19974.1). (B) A clustal w alignment was performed with the N-terminal end of human (HsTCEA1), *S. cerevisiae* (ScTFIIS), *T. brucei* (TbTFIIS1 and TbTFSIIS2-1) and *T. thermophila* SB210 (TtTFIIS1) TFIIS proteins. Identical and similar residues are shaded grey and dark respectively. (C) clustal w alignment of the C-terminal domains of human, *S. cerevisiae*, *T. brucei*, *T. thermophila* TFIIS and *Halobacterium* sp. NRC-1 TFS (HspTFS). Identical and similar residues are shaded grey and dark respectively. Asterisks and hashes indicate the position of the zinc finger cysteines and the conserved acidic residues, respectively, of domain III. The position of the LW motif is indicated as a thick grey bar.

We investigated the domain architecture of TbTFIIS1 and TbTFIIS2-1 in light of the structural and functional information available for the *S. cerevisiae* protein (Kettenberger *et al*., 2003). Both proteins have canonical domains II and III (Fig. 2A and B; Fig. S1). Because the domains III from both *T. brucei* proteins also contain the four-cysteine zinc finger motif and the aspartate-glutamate amino acid residue pair, there is no apparent impairment to these proteins functioning as TFIIS factors. The functional relevance of domain I for TFIIS *in vivo* appears to be species-specific (Nakanishi *et al*., 1995; Ling *et al*., 2006). TbTFIIS2-1 contains a conserved domain I including an LW motif. In TbTFIIS1, domain I is either entirely absent or degenerate (Fig. 2C; Fig. S1). This is also true for the *Leishmania* TFIIS1-1 paralogues (LmjF24.0210; LinJ24_V3.0200; LbrM24_V2.0200). All of the TFIIS2-1 homologues also bear an N-terminal extension containing a PWWP domain (Fig. S2). TFIIS2-1 from trypanosomes is the first TFIIS factor described to date harbouring this domain. Interestingly, when we searched the predicted proteome of the red alga *Cyanidioschyzon merolae* with a PWWP profile-hidden Markov model, we found a putative TFIIS protein that also contained an N-terminal PWWP domain (CMT501C). Our phylogenetic analyses strongly suggest that this similar domain architecture cannot be explained by horizontal gene transfer between red algae and trypanosomes. Moreover, given the position of the red algal and trypanosomal lineages, it is most parsimonious that this similar domain architecture is the result of convergent evolution from independent events.

In addition to trypanosomes and vertebrates, we detected multiple TFIIS proteins also in two other lineages – five in *Paramecium tetraurelia* and two in *Drosophila melanogaster* (Fig. 1). Multiple gene copies in *P. tetraurelia* are common due to successive whole-genome duplications (Aury *et al*., 2006). The more canonical *D. melanogaster* TFIIS (CG3710-PA) is coded for on chromosome 2L. We identified a second homologue, *D. melanogaster* CG8117-PA, coded for by a gene on the X chromosome. Interestingly, this divergent protein does not contain a TFIIS domain I.

Our phylogenetic analysis indicates strong support for an independency of the gene duplication events resulting in the multiple TFIIS proteins that we found in *P. tetraurelia*, trypanosomes, *D. melanogaster* and vertebrates (Fig. 1; for details, see *Experimental procedures*).

### TbTFIIS1 and TbTFIIS2-1 gene expression

**Fig. 3:**
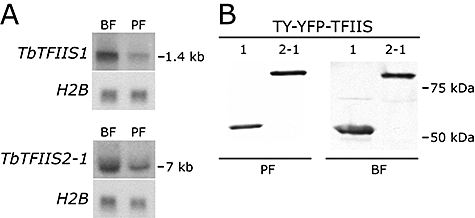
Expression of TbTFIIS1 and TbTFIIS2-1 in procyclic and bloodstream form trypanosomes. A. Northern blot analysis of total RNA extracted from procyclic (PF) and bloodstream form (BF) trypanosomes. RNA was probed with the *TFIIS1* ORF, *TFIIS2-1* ORF or *H2B* probes as indicated. Labelled bars indicate mRNA size. B. Immunoblot analysis of whole-cell extracts from procyclic and bloodstream form cells expressing TY-YFP-tagged TFIIS1 or TFIIS2-1 respectively. Proteins were detected with monoclonal BB2 (anti-TY) antibody.

We investigated the expression of *TFIIS1* and *TFIIS2-1* in *T. brucei*. Both mRNAs were detected by Northern blot analysis in bloodstream form and procyclic cells (Fig. 3A). Interestingly, while the *TbTFIIS1* mRNA was about 800 nucleotides longer than its open reading frame (ORF), the size of the *TbTFIIS2-1* mRNA was estimated to be around 7 kb, which is approximately 5.5 kb larger than its ORF. As the Tb927.2.3610 and the *TbTFIIS2-2* ORFs are located about 2.2 kb upstream and 4.8 kb downstream of the *TbTFIIS2-1* ORF, respectively, it suggests that the *TbTFIIS2-1* 3′ UTR is at least around 3.5 kb long.

We then studied the expression of the two TbTFIIS genes at the protein level. Because attempts to generate anti-TbTFIIS antibodies have proven unsuccessful so far, we endogenously tagged the N-termini of TbTFIIS1 and TbTFIIS2-1 with TY-YFP in procyclic and bloodstream form parasites. Tagging of the C-termini was avoided in order not to interfere with the structure of this part of the protein that might be crucial for its interaction with pol II. The correct tagging and expression of the fusion proteins were controlled for by genomic polymerase chain reaction (PCR) (data not shown). Immunoblot analysis showed that both proteins were expressed in both procyclic and bloodstream form cells (Fig. 3B)

### TbTFIIS1 and TbTFIIS2-1 show similar but distinct distribution patterns in the nucleus

**Fig. 4:**
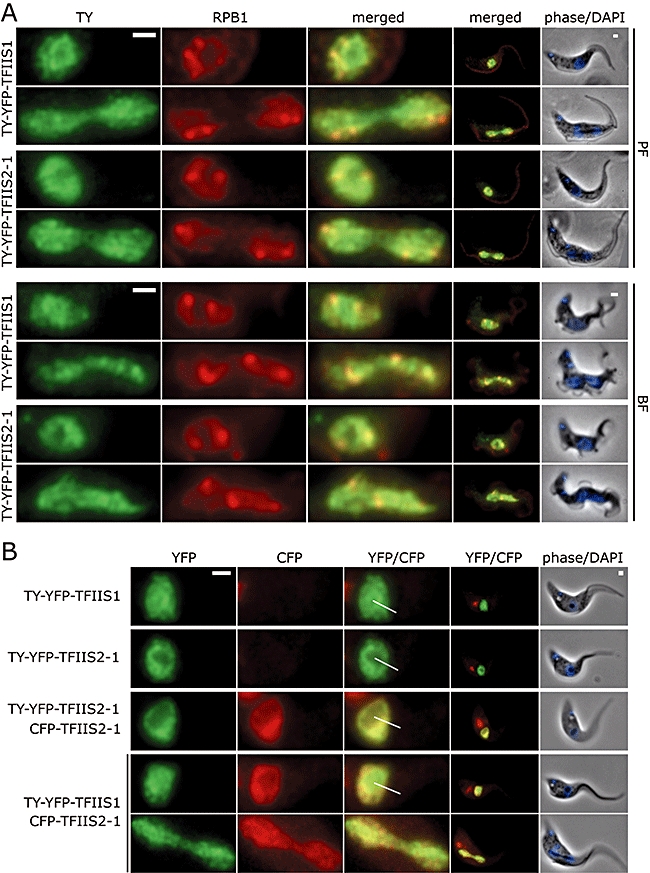
Subcellular localization of TbTFIIS1 and TbTFIIS2-1 in procyclic and bloodstream form trypanosomes. A. Subnuclear localization of TY-YFP-tagged TbTFIIS1 and TbTFIIS2-1, compared with localization of RPB1, the largest subunit of RNA polymerase II. Procyclic cells (PF) and bloodstream form cells (BF) labelled with BB2 (anti-TY) monoclonal antibody and anti-RPB1 antiserum. In both life cycle stages, both proteins were detected in a pattern of small foci heterogeneously distributed throughout the nucleus. They exhibited partial colocalization with the low-intensity nucleoplasmic RPB1 signal. The TFIIS proteins, however, were not enriched in the RPB1 macro-foci. Bars, 1 μm. B. Comparison of the subnuclear localizations of TY-YFP–TFIIS1 and CFP–TFIIS2-1 in procyclic cells. YFP fluorescence is pseudo-coloured in green, CFP in red (see *Experimental procedures*). There is a cytoplasmic signal in the CFP channel in all cells which is caused by autofluorescence. The left end of the thin white line points at the central region of the nucleoli in the overlay column. The TY-YFP–TFIIS1 fluorescence was more concentrated towards the periphery of the nucleolus and had signal inside the nucleolus, while the CFP–TFIIS2-1 fluorescence was clearly excluded from the nucleolus. Bars, 1 μm.

Both TY-YFP–TFIIS1 and TY-YFP–TFIIS2-1 were found to localize specifically to the nuclei of procyclic and bloodstream form cells throughout the full cell cycle (Fig. 4A). The observed patterns were roughly similar and consisted of small foci that were heterogeneously distributed throughout the nucleoplasm. For both proteins, we noticed a tendency to be excluded from areas which were strongly stained with DAPI and might therefore correspond to heterochromatin (Fig. S3). Looking at a large number of cells, we suspected the presence of subtle differences between the nuclear distributions of TbTFIIS1 and TbTFIIS2-1. Therefore, we coexpressed TY-YFP–TFIIS1 with CFP–TFIIS2-1. Correct tagging was controlled for by immunoblot analysis (data not shown). Comparing the fluorescence of the fusion proteins we found that TY-YFP–TFIIS1 fluorescence was more concentrated towards the periphery of the nucleolus and had signal in the nucleolus, while the CFP–TFIIS2-1 fluorescence was clearly excluded from the nucleolus (Fig. 4B). A control cell line was generated in which both alleles of TbTFIIS2-1 were labelled with TY-YFP and CFP respectively. Thus, we ensured that the two tags did not differentially influence the localization of the protein. Overall, these results indicate only a partial colocalization of TbTFIIS1 and TbTFIIS2-1 within procyclic nuclei.

The most dramatic difference in domain architecture between the two proteins is the presence of an N-terminal PWWP domain in TFIIS2-1 only. As this domain has been shown to be important for targeting of DNA methyltransferases to heterochromatin (Chen *et al*., 2004; Ge *et al*., 2004), we tested whether it had any influence on the subnuclear localization of TbTFIIS2-1. We therefore replaced the 77 N-terminal amino acids of one endogenous allele of TbTFIIS2-1 with TY-YFP in procyclic *T. brucei* (Figs S2 and S4A). This protein was still transported into the nucleus, but no difference was seen between the subnuclear pattern of this truncated and the full-length TY-YFP–TFIIS2-1 fusion protein (Fig. S4B). The PWWP domain is therefore not required for the nuclear or subnuclear localization patterns of TbTFIIS2-1.

**Fig. 5:**
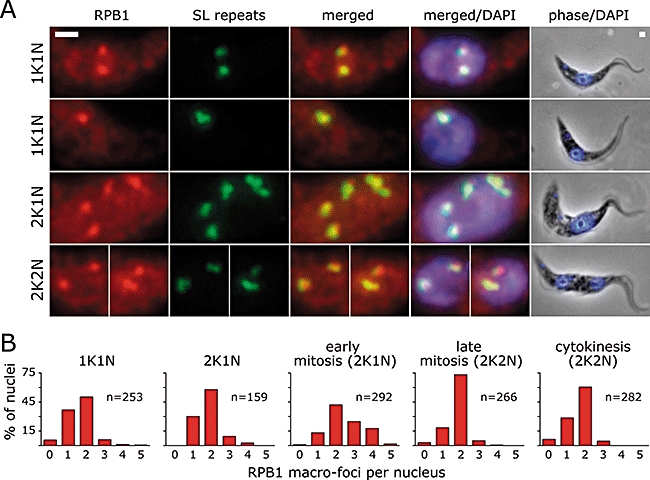
Colocalization of *SL RNA* loci with the RPB1 macro-foci. A. DNA FISH of the *SL RNA* repeat with a digoxigenin-labelled *SL RNA* repeat probe combined with immunofluorescence using rabbit anti-RPB1 antiserum on procyclic cells. Almost all 1K1N cells display either one or two RPB1/*SL RNA* foci. During nuclear division, up to four foci can be found. Bars, 1 μm. B. Quantification of the number of RPB1 macro-foci during the cell cycle. Procyclic cells were labelled in immunofluorescence with rabbit anti-RPB1 antibody and mouse monoclonal KMX (anti-beta-tubulin). Cells were divided into five cell cycle categories: 1K1N, 2K1N without nuclear beta-tubulin spindle, 2K1N with spindle (early mitosis 2K1N), 2K2N with spindle (late mitosis 2K2N) and 2K2N without spindle (cytokinesis 2K2N). RPB1 macro-foci per nucleus were counted. In 1K1N, more cells display two RPB1 macro-foci than only one. During nuclear division (2K1N with spindle and 2K2N with spindle), up to four RPB1 macro-foci can be observed.

As TFIIS acts to relieve arrested pol II in other eukaryotes studied, we investigated whether the tagged TbTFIIS proteins would colocalize with the largest subunit of this enzyme complex, RPB1. By immunofluorescence using an anti-RPB1 antiserum, we observed prominent nuclear macro-foci in addition to a basic nucleoplasmic RPB1 pattern (Figs 4A and 5A; Das *et al.*, 2006). In *T. cruzi*, RPB1 has been found to be enriched in a single nuclear focus which represents the site of active SL RNA transcription (Dossin and Schenkman, 2005). By DNA fluorescence *in situ* hybridization (FISH) against the full-length *SL RNA* repeat the RPB1 macro-foci did indeed colocalize with the SL RNA alleles in procyclic *T. brucei* (Fig. 5A). However, in contrast to *T. cruzi*, most 1K1N cells had two RPB1 macro-foci (Fig. 5B). During nuclear division, up to four macro-foci were observed per nucleus (17.8%, *n* = 266), corresponding to all four *SL RNA* repeat alleles present after S-phase (Fig. 5A and B). The TY-YFP–TFIIS1 and –TFIIS2-1 foci colocalized partially with the basic nucleoplasmic RPB1 signal. However, TFIIS was not noticeably enriched at the prominent RPB1 macro-foci. This might indicate that transcription of the short and monocistronic SL RNA precursor does not normally require association with TFIIS.

Neither TFIIS protein was found to be restricted to a pattern characteristic of pol I in bloodstream form cells (Navarro and Gull, 2001), suggesting a lack of specificity for that enzyme complex.

### Simultaneous reduction of both TFIIS proteins impairs trypanosome growth

**Fig. 6:**
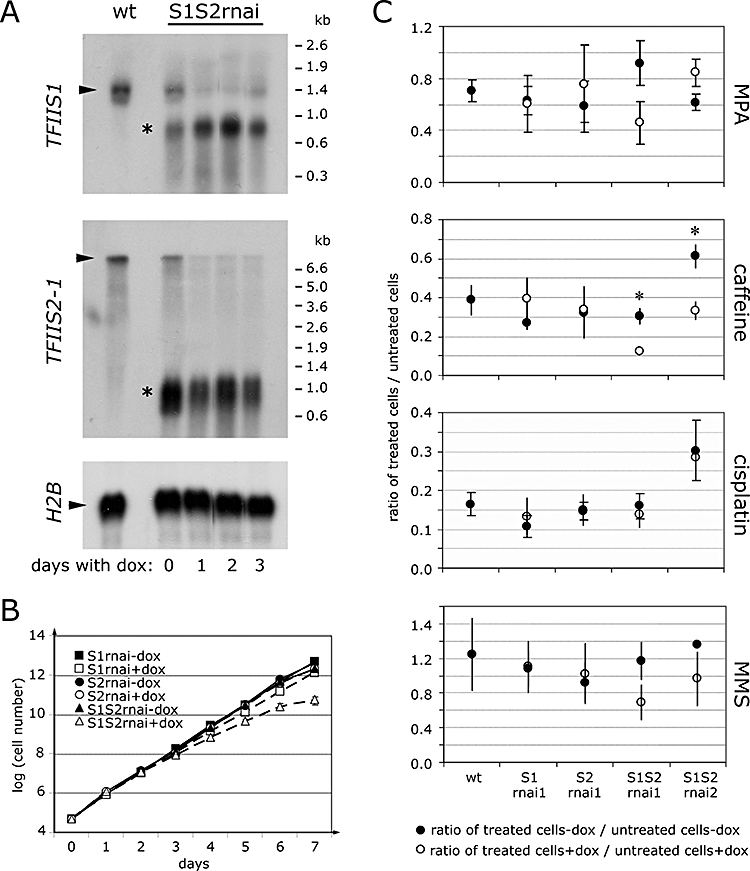
RNAi of *TbTFIIS1* and *TbTFIIS2-1* in bloodstream form trypanosomes. A. Northern blot analysis of total RNA extracted from wild type (wt) and *TbTFIIS1* and *TbTFIIS2-1* double knock-down clone 1 (S1S2rnai) cells at days 0–3 after doxycycline induction. The RNA was probed with the *TFIIS1* ORF, *TFIIS2-1* ORF or *H2B* probes as indicated. Arrows and asterisks indicate the mRNA and the induced double-stranded RNA respectively. RNA molecular weight marker sizes are indicated on the right. B. Growth curves of the single *TFIIS1* (S1rnai; squares) and *TFIIS2-1* (S2rnai; circles) knock-down and double *TFIIS1/TFIIS2-1* (S1S2rnai1; triangles) knock-down clones. Dark and light symbols correspond to uninduced and doxycycline-induced cells respectively. Counts were performed on culture triplicates. C. Toxicity of MPA, caffeine, cisplatin and MMS on wild-type cells, S1rnai, S2rnai, S1S2rnai1 and S1S2rnai2 clones. Diagrams present the ratio between drug-treated and control cell numbers monitored at day 4 (see *Experimental procedures*). Counts were performed on culture triplicates. Mean values obtained for uninduced and doxycycline-induced cells are indicated as dark and open circles respectively. Asterisks indicate significant difference between the values (*P < 0.01; Student's *t*-test).

To evaluate the functional importance of the *T. brucei* TFIIS proteins for cell growth, transgenic bloodstream form parasites expressing tetracycline-inducible RNA interference (RNAi) constructs were generated in order to knock down each of them. The S1rnai and S2rnai transgenic trypanosome clones were selected which contained inducible TbTFIIS1 or TbTFIIS2-1 RNAi constructs, respectively, as well as S1S2rnai clones containing both TbTFIIS1 and TbTFIIS2-1 RNAi constructs. The efficiency of the *TbTFIIS1* or *TbTFIIS2-1* mRNA knock-down upon double-stranded RNA induction was checked and found to be indistinguishable in the single RNAi knock-down cell lines compared with the double RNAi knock-down cell lines (data not shown). Induction of the TbTFIIS1 and TbTFIIS2-1 RNAi constructs lead to a reduction of the corresponding mRNA within 24 h to about 10% of wild-type levels, and these mRNA levels were maintained for at least 3 days (Fig. 6A). These levels of reduction of *TbTFIIS1* or *TbTFIIS2-1* mRNA were comparable in transgenic procyclic form trypanosomes expressing the same TbTFIIS1 or TbTFIIS2-1 RNAi constructs respectively (data not shown). In both life cycle forms, single knock-down of TbTFIIS1 or TbTFIIS2-1 did not significantly alter cell growth as compared with non-induced cells (Fig. 6B and data not shown). On the contrary, the growth of bloodstream form trypanosomes expressing both TbTFIIS1 and TbTFIIS2-1 RNAi constructs was impaired after 3 days of culture (Fig. 6B). This limited impairment of cell growth suggests that TFIIS function is either dispensable or sufficiently provided by the low amounts of TbTFIIS in these cells (Fig. 6A).

We then asked if these RNAi cell lines were more susceptible to pharmacological insults affecting transcription. Cells were therefore treated with MPA (which reduces the availability of intracellular nucleotide levels and thus impairs transcription elongation and processivity), caffeine [which has been shown to negatively affect the growth of a TFIIS deletion mutant of budding yeast (Wery *et al*., 2004)], cisplatin and MMS (which might also interfere with the elongation phase of transcription). Figure 6C shows the ratio between the cell numbers evaluated after 4 days of *in vitro* cultivation in the presence and absence of the drugs. At the dose used, MPA, caffeine and cisplatin were found to alter wild-type trypanosome growth.

The only drug with a specific growth effect on RNAi induction was caffeine. Growth of TFIIS double knock-down cells was reduced in both S1S2rnai clones (Fig. 6C). TFIIS was recently reported to increase resistance of budding yeast to oxidative stress (Koyama *et al*., 2003) and to aid pol II in bypassing of oxidative DNA damages in the human system (Charlet-Berguerand *et al*., 2006; Kuraoka *et al*., 2007). However, we did not find any differences of the sensitivity of bloodstream form TFIIS single and double knock-down cells to 100 μM H_2_O_2_ treatment compared with control cells (data not shown).

### Expression of one TbTFIIS protein is necessary and sufficient for growth of procyclic cells

**Fig. 7:**
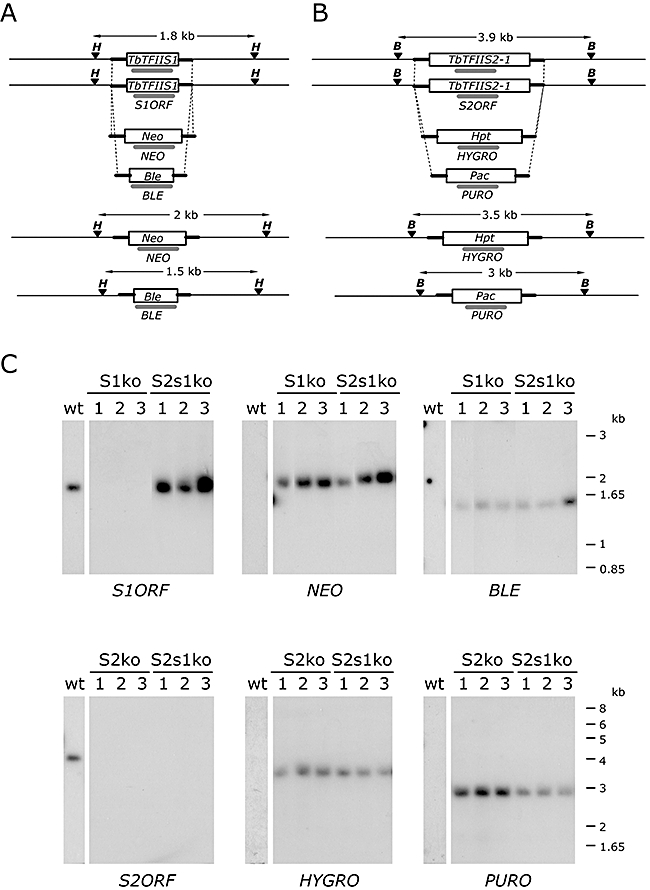
Southern blot analysis of *TbTFIIS1* and *TbTFIIS2-1* knockout procyclic forms. A and B. Schematic drawing of the *TbTFIIS1*, *Neo* and *Ble* (A) and *TbTFIIS2-1*, *Hpt* and *Pac* (B) genes indicating the relative positions of the HpaI (*H*) or Bsp1407I (*B*) restriction sites. The transfected PCR fragments cover the *Neo*, *Ble*, *Hpt* or *Pac* resistance marker genes flanked by 100 bp immediately preceding the start and stop codon of the *TbTFIIS1* or *TbTFIIS2-1* ORF. The homologous regions are thick-lined. The probes hybridizing to *TbTFIIS1* (*S1ORF*), *TbTFIIS2-1* (*S2ORF*), *Neo* (*NEO*), *Ble* (*BLE*), *Hpt* (*HYGRO*) or *Pac* (*PURO*) sequences are labelled by a thick grey line. The expected size of the restriction fragments is indicated. C. Southern blot analyses of the DNA extracted from the wild-type trypanosomes (wt), *TbTFIIS1* single knockout (S1ko), *TbTFIIS2-1* single knockout (S2ko) and attempted double knockout (S2s1ko) clones 1, 2 and 3 respectively. The DNA was digested with HpaI (S1ko, S2s1ko) or Bsp1407I (S2ko, S2s1ko) restriction enzymes, separated by electrophoresis and hybridized with the probes as indicated.

**Fig. 8:**
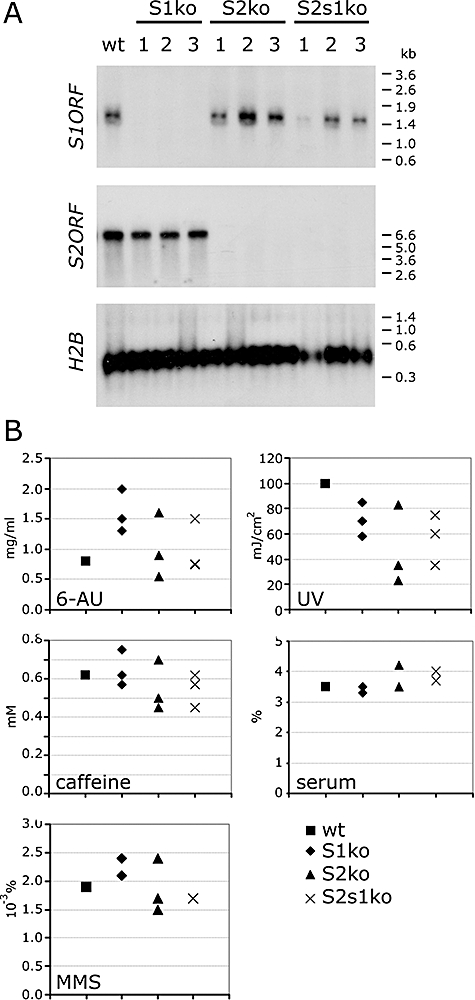
Characterization of the *TbTFIIS1* and *TbTFIIS2-1* knockout procyclic cells. A. Northern blot analysis of the total RNA extracted from wild-type trypanosomes (wt), TFIIS1 knockout (S1ko), TFIIS2-1 single knockout (S2ko) and attempted double knockout (S2s1ko) clones 1, 2 and 3. The RNA was probed with the *TFIIS1* (*S1ORF*), *TFIIS2-1* (*S2ORF*) or *H2B* probes as indicated. RNA molecular weight marker sizes are indicated on the right. B. Values of the cell number-reducing dose 50 upon 6-AU, caffeine, MMS, UV irradiation and low serum concentration treatment on wild type (square), *TbTFIIS1* single knockout (S1ko; diamond), *TbTFIIS2-1* single knockout (S2ko; triangle) or double knockout (S2s1ko; cross) clones 1, 2 and 3. Values were calculated as indicated in *Experimental procedures*. Strains are plotted on the *x*-axis and each point corresponds to one clone. Scale units are indicated on the left.

In order to test whether the lack of a growth defect in the single TFIIS knock-down cells was caused by residual amounts of protein, constructs were designed in order to knock out *TbTFIIS* genes (Fig. 7A and B). These constructs were transfected into procyclic form cells. Southern blot analyses indicated that both the *TbTFIIS1* and *TbTFIIS2-1* alleles were, respectively, replaced by the *Neo*/*Ble* (S1ko clones) and *Hpt*/*Pac* (S2ko clones) genes as predicted (Fig. 7B). Northern blot analyses of total RNA of S1ko or S2ko clones using the *S1ORF* and *S2ORF* probes confirmed the absence of *TbTFIIS1* or *TbTFIIS2-1* mRNA respectively (Fig. 8A). This analysis also showed that the *TbTFIIS2-1* or *TbTFIIS1* mRNA abundance was not affected in S1ko or S2ko cells respectively. Despite the complete lack of one of the respective TbTFIIS protein, the rate of cell growth was not affected. The population doubling time of the S2ko clones was indistinguishable from the 12 ± 0.8 h measured for the wild-type strain, and the doubling time of the S1ko clones was 12.8 ± 1.2 h.

These results show that under optimal growth conditions, each TbTFIIS protein is non-essential. This was possibly due to a redundancy between the two proteins. To evaluate this hypothesis, the S2ko procyclic cell population was subsequently super-transfected with constructs targeting the *TbTFIIS1* gene. After selection, the resistant population was cloned and the gDNA of the generated S2s1ko clones was analysed by Southern blot. As expected, the pattern observed with the *S2ORF*, *HYGRO* and *PURO* probes was identical to that observed in the S2ko clones. However, although the *NEO* and *BLE* probes detected the expected restriction fragments, the *S1ORF* probe still hybridized to a *TbTFIIS1* allele, demonstrating that this attempt to generate double knockout cells failed (Fig. 7C). Indeed, Northern blot analysis of total RNA of the S2s1ko clones using the *S1ORF* probe showed that *TbTFIIS1* mRNA expression was not affected (Fig. 8A).

In an alternative strategy, the S1ko cells were super-transfected with the constructs targeting the *TbTFIIS2-1* gene. While the first allele could efficiently be deleted as shown by the analysis of the generated resistant population, attempts to knock out the second allele did not allow the selection of recombinant trypanosomes resistant to all four drugs (data not shown). This strongly suggests that it is not feasible to generate cells with both *TbTFIIS* genes knocked out and that the presence of either TbTFIIS1 or TbTFIIS2-1 is essential for normal growth of procyclic-form trypanosomes.

The sensitivity of these recombinant procyclic cells to stress conditions was evaluated as follows. The S1ko, S2ko and S2s1ko clones were cultivated for 48 h in the presence of a broad range of concentrations of 6-AU, caffeine or MMS. UV exposure and low serum concentrations were also tested on these clones, as exposure of DNA to UV produces pyrimidine bridges that impair transcription. We evaluated the drug concentration, UV dose and low serum concentration leading to a 50% reduction of cell number at 48 h (RCD50, reduction of cell number dose 50). None of the clones were sensitized to 6-AU, caffeine, MMS or low serum concentration (Fig. 8B). Although they all presented a slightly increased sensitivity to UV, these differences are not statistically significant (Fig. 8B).

## Discussion

Many features of nuclear transcription of trypanosomes are unusual compared with other well-studied eukaryotic organisms. Pol II transcription of protein-coding genes is polycistronic, and pol I in *T. brucei* not only transcribes rRNA precursors, but also long clusters of protein-coding genes. Analyses of genome sequences and proteomic studies have shown that a number of the basal eukaryotic transcription initiation factors are either absent or very divergent in trypanosomes.

In order to investigate the relevance of transcription elongation factors for trypanosome transcription, we conducted a bioinformatic search for TFIIS proteins in *T. brucei*. We identified two potentially functional proteins, TbTFIIS1 and TbTFIIS2-1. Both were expressed in procyclic and bloodstream form cells. Endogenously tagged versions of the proteins were found in a heterogeneously distributed pattern of nuclear foci throughout the cell cycle. We were unable to detect any specific association of tagged versions of both proteins with specific genomic regions by chromatin immunoprecipitation (ChIP) (P. Uzureau, unpublished results). However, this is reminiscent of observations in *S. cerevisiae*, where TFIIS proteins could also not be detected by ChIP on the *GAL1* ORF when its transcription was induced under normal conditions (Pokholok *et al*., 2002). Neither single knock-down nor individual knockout of TbTFIIS1 and TbTFIIS2-1 impaired trypanosome growth. A mild growth phenotype was observed, however, in bloodstream form double knock-down cells. Importantly, we failed to produce procyclic cells with a knockout of both TFIIS proteins, although different strategies were attempted. This indicates that TbTFIIS1 and TbTFIIS2-1 provide a redundant function, which is essential for cell growth. This is very different from the situation in budding yeast cells, which do not require TFIIS for growth under normal conditions (Nakanishi *et al*., 1992).

We also tested the effects of drugs and conditions which potentially interfere with transcription on our TFIIS knock-down and knockout *T. brucei* cells. We observed no increased sensitivity to 6-AU or to MPA. This was unexpected, as these drugs are thought to increase the requirement for transcription elongation factors by pol II and in addition TFIIS deletion in *S. cerevisiae* cells results in highly increased drug sensitivity. Also, in a *T. brucei gambiense* strain resistant to MPA, IMPDH was found to be overexpressed by sixfold (Wilson *et al*., 1994). This indicates that the well-studied mode of action of MPA, inhibition of IMPDH, also applies to trypanosomes. Interestingly, while wild-type budding yeast grows unaltered in 100 μg ml^−1^ 6-AU, our study demonstrated that merely 1 μg ml^−1^ inhibited growth of procyclic *T. brucei* cells, demonstrating a higher basal sensitivity of this organism for 6-AU. A significant increase in sensitivity to caffeine was found for TFIIS double knock-down bloodstream form cells. Similarly, growth of TFIIS knockout budding yeast was found to be impaired in the presence of caffeine (Wery *et al*., 2004). Furthermore, yeast mutants of other proteins putatively involved in transcription elongation were hypersensitive to this drug (Frohloff *et al*., 2001; Kim *et al*., 2004). Due to the multiple effects of caffeine on cellular pathways, it is, however, not possible to interpret this result as clear evidence for the involvement of TbTFIIS in transcription.

Despite roughly similar patterns, we discovered that differentially tagged TbTFIIS1 and TbTFIIS2-1 show distinct distributions in procyclic nuclei. It seems plausible to speculate that the divergent domain architectures are responsible for these differences. In contrast to TbTFIIS1, TbTFIIS2-1 contains a TFIIS domain I, including the LW-motif. Although this sequence has been shown to be important for the import of mammalian proteins into the nucleus (Ling *et al*., 2006), this cannot be true for TbTFIIS1, because it is found in the nucleus despite the absence of this domain. Nuclear transport of *S. cerevisiae* TFIIS relies on Kap119p (Albertini *et al*., 1998). No Kap119p orthologue was identified in trypanosomes and the trypanosome members of the Kap family display more sequence similarity with the human than the budding yeast nuclear transporters (P. Uzureau, unpublished results). Domain I might, however, be required for the exact subnuclear distribution of TbTFIIS2-1.

PWWP domains are found in many nuclear proteins involved in transcriptional regulation and chromatin organization. To date, no TFIIS factor including a PWWP domain has been described. We could, however, not only identify TFIIS proteins with an N-terminal PWWP domain in trypanosomes, but also, and probably resulting from an independent evolutionary event, in a red alga. We found this domain in only two other *T. brucei* protein sequences (TbTFIIS2-2 and Tb927.7.2520). As PWWP domains are involved in targeting of DNA methyltransferases to pericentric heterochromatin, most likely via protein–protein interactions (Chen *et al*., 2004; Ge *et al*., 2004), we tested whether the PWWP domain in TbTFIIS2-1 has major influence on its subnuclear localization. However, no difference was detected between the localization pattern of ΔPWWP TbTFIIS2-1 compared with the full-length protein. Pull-downs using TAP-tagged TFIIS2-1 did not identify any specific interaction partners (P. Uzureau unpublished results). Although the distinct subnuclear distributions and the divergent domain architectures might imply additional, non-redundant functions for one or both of the two TbTFIIS proteins, these functions must be non-essential under normal conditions, because both single knockout cell lines were unaffected.

In addition to the expansion of TFIIS genes in trypanosomes, our TFIIS data set revealed the presence of multiple genes also in the ciliate *P. tetraurelia*, in *D. melanogaster* and in vertebrates (represented by *Takifugu rubripes* and *Homo sapiens*). Our phylogenetic analyses show that these four expansions were evolutionary independent. A non-redundancy for the three vertebrate TFIIS proteins has been demonstrated by *TCEA1* knockout in mice leading to embryonic lethality due to a failure of definitive haematopoiesis (Ito *et al*., 2006). In *P. tetraurelia*, multiple copies of many genes have been retained after several consecutive whole-genome duplication events, mostly rather because of dosage constraints than functional innovation (Aury *et al*., 2006). Without experimental data, however, it is impossible to judge whether the five proteins we identified harbour redundant functions. We showed that despite sequence divergence, TbTFIIS1 and TbTFIIS2-1 are functionally redundant. Still, it appears unlikely that genes coding for both proteins have been maintained due to expression-level constraints, because both single knockout cell lines grow normally and do not exhibit compensatory increase of mRNA of the respective other protein. Possibly, separate characteristics of the two proteins become critical for survival under conditions that we were not able to simulate *in vitro*.

Comparative localization of tagged TbTFIIS1 and TbTFIIS2-1 demonstrated a possible association of these proteins with pol II. Despite this, it remained unclear whether any of the TFIIS foci represent sites of active transcription or merely storage sites. In amphibian oocytes, TFIIS has been found concentrated in Cajal bodies (Smith *et al*., 2003). The presence of these or analogous nuclear bodies has not been shown in trypanosomes. Despite colocalization with the basic nucleoplasmic signal of RPB1, TbTFIIS was not enriched at the RPB1 macro-foci. Using FISH, we showed that these foci colocalize with the *SL RNA* loci and therefore represent the sites of SL RNA transcription in *T. brucei*. In contrast to the situation described in *T. cruzi* (Dossin and Schenkman, 2005), most 1K1N cells exhibited two RPB1 macro-foci instead of only one – presumably corresponding to the two alleles of the locus in the diploid cells. The lack of enrichment of TbTFIIS at the RPB1 foci suggests that they might not be required for transcription of the short, monocistronic SL RNA precursor. No restriction of any of the TFIIS proteins to a pol I-specific localization pattern was detected (Navarro and Gull, 2001). As it is difficult to envisage the monoallelic transcription of the long ES clusters of protein-coding genes by pol I without the aid of elongation factors to reduce pausing and relieve arrest, we believe that different, still unidentified factors in *T. brucei* must be responsible for these functions.

## Experimental procedures

### Bioinformatic and phylogenetic analysis

A data set of TFIIS domain II- and domain III-containing proteins was produced by iterative profile-hidden Markov model-based amino acid sequence searches. In order to gather sequences for a seed alignment, the *S. cerevisiae* Dst1p sequence (NP_011472.1) was used to query the NCBI nr database with blastp (Altschul *et al*., 1997). Several hit sequences with expectation values lower than 10^−20^ from evolutionary distant organisms were aligned using the program mafft (Katoh *et al*., 2002). The resulting multiple sequence alignment was manually edited to well aligning sequence blocks and used to generate a profile-hidden Markov model with hmmer 2.3.2 (Eddy, 1998; Devaux *et al*., 2007). This model was then used to search the predicted proteomes of two archaeal and 16 eukaryotic, evolutionary diverse organisms (see Table S1). From the resultant hits with an expectation value lower than 10^−5^ a new multiple sequence alignment was built. This alignment was trimmed to well aligning sequence blocks and sequences lacking convincing similarity to either TFIIS domain II or domain III or not containing the conserved aspartate/glutamate amino acid pair in domain III were discarded (all archaeal sequences containing domain III were kept). Highly similar sequences (identity > 90%) were also removed in order to prevent excessive bias in the profile-hidden Markov model towards redundant sequences in particular predicted proteomes. Hits from this search were taken as a new seed and the whole process was repeated until no further additional sequences were found, resulting in a data set of 28 eukaryotic TFIIS domain II- and domain III-containing proteins from all 16 organisms and one TFIIS domain III-containing protein from each of the two archaeal predicted proteomes.

Multiple sequence alignments in Fig. 2B and C and Fig. S2 were generated by clustal w (Thompson *et al*., 1994), the ones in Fig. S1 with MAFFT (Katoh *et al*., 2002).

The final trimmed and edited alignment of the 30 sequences in the data set was composed of 234 characters. Based on this alignment, Bayesian maximum-likelihood trees were inferred using MrBayes v3.1.2 (Ronquist and Huelsenbeck, 2003) with a gamma-distributed substitution rate variation approximated by four discrete categories with shape parameter estimated from the data and the WAG substitution rate matrix. One hundred replicate Bayesian tree inferences were produced with character re-sampling and used to estimate the bootstrap support for the inferred topologies. Furthermore, bootstrap support was also estimated using 100 replicate maximum-likelihood trees generated with phyml (Guindon and Gascuel, 2003) and 1000 replicate trees were made by a neighbour-joining approach using paup*4.0b10 (Swofford, 2003).

### Cell culture

Procyclic form cells from the *T. brucei* EATRO 1125 strain were grown at 27°C in SDM79 medium (Brun and Jenni, 1977) supplemented with 15% fetal bovine serum. Bloodstream form ‘single-marker’ (Wirtz *et al*., 1999) cells were grown at 37°C in 5% CO_2_ in HMI-9 medium (Hirumi and Hirumi, 1989) supplemented with either 20% fetal bovine serum or 10% fetal bovine serum with 10% serum plus. Electroporation and selection procedures were conducted as described (Devaux *et al*., 2006).

### RNA interference (RNAi)

The full ORF of TbTFIIS1 (Tb11.02.2600) and a fragment ranging from nucleotides 25 to 859 of TbTFIIS2-1 (Tb927.2.3580) were amplified by PCR using specific pairs of primers and, respectively, inserted into the p2T7-177 (Wickstead *et al*., 2002) or pZJM (Wang *et al*., 2000) vectors allowing the inducible expression of double-stranded RNA, by using appropriate restriction sites. These vectors were linearized with the NotI restriction endonuclease and then transfected successively into bloodstream form ‘single-marker’ cells as described previously (Wirtz *et al*., 1999). Stable transformants were selected with 1 μg ml^−1^ hygromycin (InvivoGen) and/or phleomycin (InvivoGen) respectively. After selection of resistant populations or clones, RNAi was induced by addition of 1 μg ml^−1^ doxycycline (Duchefa) to the culture medium. Cloning of bloodstream form cells was performed either by limiting dilution on 96-well plates or by plating cells on agarose (Carruthers and Cross, 1992). Northern blots were performed according to standard procedures.

### Expression of chimeric proteins

For the generation of cells expressing chimeric proteins consisting of an N-terminal TY epitope (Bastin *et al*., 1996) and enhanced-yellow fluorescent protein (YFP) from the endogenous loci of the TFIIS genes, fragments of 300–400 nucleotides of the 5′ end of the ORF and the 3′ end of the respective upstream intergenic region of the *TbTFIIS1* (Tb11.02.2600) and *TbTFIIS2-1* (Tb927.2.3580) genes were amplified by PCR using the following primers: 2600_ORF_22F (5′-GCt cta gaG GTA AGG ATT CCA ATG ATG GGA-3′), 2600_ORF_22R (5′-CCc tcg agC CTC TCT TTC ATA CGC ACC AAA-3′), 2600_UTR_F (5′-AGc tcg agC GTA TCA CGA GGA TGT GGA GTC-3′) and 2600_UTR_R (5′-TCg gat ccC ACG TAC GAA CGC TGA GAA GG-3′) for *TbTFIIS1*, and 3580_ORF_F (5′-GCt cta gaC TTC AAG AAA GAG TAT TTC ATA TTA AC-3′), 3580_ORF_R (5′-CCc tcg agA AAC GCC CTC TGT GAT GTT C-3′), 3580_UTR_F (5′-CCc tcg agC CTT TAA TGT TTT CCG CAC TTC C-3′) and 3580_UTR_R (5′-CGg gat ccG CTT TCC TCC ACC CTT TCT TTT C−3′) for *TbTFII2-1*. The ORF and intergenic region fragments of each gene were ligated together into the endogenous locus tagging vector pEnT6B-Y (Kelly *et al*., 2007) utilizing the XbaI and BamHI restriction sites and generating the vectors pEnT6B-Y:TFIIS1 and pEnT6B-Y:TFIIS2-1 respectively. pEnT6B-Y:TFIIS2-1ΔPWWP was made by replacing the TFIIS2-1 ORF fragment in pEnT6B-Y:TFIIS2-1 with a PCR fragment amplified from genomic DNA with the primers 3580_PWWP_F (5′-GCt cta gaT CAT CTG AGA AAG CCG TCA C-3′) and 3580_PWWP_R (5′-AAc tcg agT TTG TGC GCA AAA GCT GCC G-3′) using the XbaI and XhoI sites. In order to generate cells expressing N-terminal CFP chimeric proteins from the endogenous loci of the *TbTFIIS2-1* gene, the ORF of cerulean fluorescent protein (CFP) was amplified from pEnT6P-C (Kelly *et al*., 2007) by PCR using primers HindATGYFP_F (5′-GAa agc tt*A TG*G TGA GCA AGG GCG AGG AGC-3′) and YFPXba_R (5′-CAA Ctc tag aCT TGT ACA GCT CGT CCA TG-3′). Then, the TY-YFP fragment in the vector was replaced with this CFP fragment. The puromycin resistance marker ORF was cut out from pEnT6P-C using the restriction enzymes EcoRI and NcoI and the fragment was then used to replace the blasticidin resistance marker ORF in the vector, generating pEnT7P-C:TFIIS2-1. pEnT6B-Y:TFIIS1, pEnT6B-Y:TFIIS2-1 and pEnT7P-C:TFIIS2-1 were linearized at the XhoI restriction site and transfected into procyclic or bloodstream form Lister 427 cells following standard protocols. Stable transformants were selected for with 10 μg ml^−1^ (procyclic form) or 5 μg ml^−1^ (bloodstream form) blasticidin or 1 μg ml^−1^ puromycin respectively.

### TbTFIIS1 and TbTFIIS2-1 knockout

In order to knock out the *TbTFIIS1* gene, the *Neo* and *Ble* resistance marker genes were flanked by the 100 bp immediately upstream of the start codon and immediately downstream of the stop codon in the 5′ and 3′ UTR of the *TbTFIIS1* ORF, respectively, by PCR. PCR fragments were purified before transfection into EATRO 1125 strain procyclic cells. Stable transformants were selected with 15 μg ml^−1^ G418 (Duchefa) or 2.5 μg ml^−1^ phleomycin (InvivoGen) respectively. Cloning of procyclic cells was performed by limiting dilution on 96-well plates. To knock out the *TbTFIIS2-1* gene, the procedure was repeated using the *Hpt* and *Pac* resistance marker genes, followed by the selection of stable transformants with 25 μg ml^−1^ hygromycin(InvivoGen) or 0.1 μg ml^−1^ puromycin (Sigma) respectively. Southern and Northern blots were performed according to standard procedures.

### Immunoblot analysis, immunofluorescence, FISH and fluorescence microscopy

Immunoblot analyses using the monoclonal BB2 anti-TY antibody were conducted as previously described (Bastin *et al*., 1996).

For immunofluorescence, *T. brucei* cells were washed in phosphate-buffered saline (PBS; 137 mM NaCl, 3 mM KCl, 10 mM Na_2_HPO4, 1.8 mM KH_2_PO_4_) and allowed to settle onto glass (procyclic cells) or glutaraldehyde-derivatized silanized slides (bloodstream form cells). Subsequently, cells were fixed for 10 min in 3% (w/v) formaldehyde and the slides were washed in 0.25% (w/v) glycine in PBS. Cells were then permeabilized for 2 min with 0.1% (v/v) NP-40 in PBS and washed in 0.25% (w/v) glycine in PBS. For CFP- and YFP-fluorescence experiments, the slides were washed in PBS and mounted in Vectashield medium containing DAPI (4′,6-diamidino-2-phenylindole) (Vector Laboratories). For immunofluorescence, cells were then incubated with primary antibody [mouse monoclonal anti-TY antibody BB2 or mouse monoclonal anti-beta-tubulin antibody KMX and rabbit polyclonal anti-RPB1 antiserum – gift of Vivian Bellofatto, New Jersey Medical School, USA (Das *et al*., 2006)], diluted in PBS, for 1 h at 37°C. The slides were washed in PBS and then incubated with goat anti-mouse IgG FITC (fluorescein-isothiocyanate)-conjugated (Sigma-Aldrich) and goat anti-rabbit Alexa-Fluor 594-conjugated (Invitrogen) secondary antibodies, diluted in PBS, for 1 h at 37°C. Next, slides were mounted as described above. Images were corrected for bleed-through of DAPI signal into the CFP-fluorescence channel by colour compensation with ImageJ software (Abramoff, 2004).

For FISH, almost the entire *SL RNA* repeat was amplified by PCR from genomic DNA using the primers 5′-TTC TGG CAC GAC AGT AAA and 5′-CGG TAG ACT TAT GGG AAA C. The digoxigenin-labelled probe was generated using the Roche Nick Translation Kit supplemented with digoxigenin-dUTP (Roche). Procyclic cells were washed in PBS and allowed to settle on glass slides. Cells were fixed for 10 min with 4% (w/v) formaldehyde, 5% (v/v) acetic acid in PBS, washed with 0.25% glycine in PBS and then permeabilized for 5 min in 0.1% NP40 in PBS. After washing the slides in PBS, RPB1 immunofluorescence was performed as described above, with the addition of 100 μg ml^−1^ DNase-free RNase A to the primary antibody (Sigma-Aldrich). Next, slides were post-fixed in 3% formaldehyde in PBS for 10 min and washed in 0.25% glycine in PBS. Cells were incubated in hybridization buffer (50% formamide, 5% dextran sulphate, 25 mM sodium phosphate pH 7.0, 150 mM NaCl, 15 mM sodium citrate) for 15 min at 40°C. Then, hybridization buffer containing 50 ng of probe DNA, 12.5 μg of herring sperm DNA and 12.5 μg of yeast tRNA was added per sample. The slides were heated to 80°C for 5 min and then incubated overnight at 37°C. The slides were washed twice for 10 min in 50% formamide, 300 mM NaCl, 30 mM sodium citrate, pH 7.0 at 37°C, for 10 min in 300 mM NaCl, 30 mM sodium citrate, pH 7.0 at 55°C, twice for 20 min in 30 mM NaCl, 3 mM sodium citrate, pH 7.0 55°C, for 10 min in 600 mM NaCl, 60 mM sodium citrate, pH 7.0 and briefly in PBS. The second immunofluorescence step and slide embedding were performed essentially as above, using mouse anti-digoxigenin antibody (Roche) primary and goat anti-mouse IgG FITC-conjugated (Sigma-Aldrich) secondary antibody.

### Cytotoxicity assays

Bloodstream form trypanosomes were cultivated for 5 days in the presence of 0.2 μg ml^−1^ MPA (Sigma), 0.5 mg ml^−1^ caffeine (Janssen Chimica), 0.3 μM cisplatine (Sigma) or 0.3*10^−3^ MMS (Sigma). Culture medium and drugs were changed daily and trypanosomes were always maintained below 1*10^6^ cells ml^−1^. Assays were performed in triplicates. Daily counted cell density was used to calculate the cell number that emerged from 5 days of cultivation of 1*10^5^ cells (Fig. 6B). The cell number calculated in the presence of MPA, caffeine, cisplatine or MMS was then divided by the corresponding cell number calculated without drug to establish the drug/no drug ratios. The same experimental procedure was then repeated after addition of 1 μg ml^−1^ doxycycline (Duchefa) to the culture medium.

Exponentially growing procyclic cell cultures were diluted to 1*10^6^ cells ml^−1^ and loaded in 96-well plates. Cells were either incubated with 3*10^−3^ to 10 μg ml^−1^ 6-AU (Sigma), 1*10^−3^ to 3 mg ml^−1^ caffeine (Janssen Chimica), 3*10^−6^ to 0.01% MMS (Sigma), treated with UV doses ranging from 0.1 to 100 mJ cm^−2^ or cultivated in 0.07–15% serum concentration. Assays were performed in triplicates. Following incubation for 48 h at 27°C, 20 μl of ‘Celltiter 96 aqueous one solution cell proliferation assay’ (Promega) was added per 100 μl of culture medium and plates were further incubated for 4 h at 37°C. The absorbance of each well was determined at 490 nm, and resultant data were used to calculate the reduction of cell number by comparison with untreated controls. The cell number-reducing dose 50 was graphically evaluated on diagrams plotting the calculated growth inhibition against the corresponding drug concentration.

Figure S5 shows the accuracy of the Celltiter 96 assay for procyclic forms. In brief, the linear relationship between the 490 nm OD and the cell density was checked for all tested populations as follows: cell concentrations of serially diluted cultures were determined using a Neubauer-counting chamber and plotted against the 490 OD values obtained from the Celltiter 96 assay. A linear relationship between parasite density and Celltiter 96 assay values was observed within the 5*10^5^ to 2*10^7^ parasites ml^−1^ concentration range. Dead trypanosomes were not detected by this method (data not shown).

## Supplementary material

This material is available as part of the online article from: http://www.blackwell-synergy.com/doi/abs/10.1111/

j.1365-2958.2008.06348.x

(This link will take you to the article abstract).
