# Supplementary material for: Identification and characterization of two trypanosome TFIIS proteins exhibiting particular domain architectures and differential nuclear localizations
Source: Mol Microbiol. 2008 Jul 15;69(5):1121–36. doi: 10.1111/j.1365-2958.2008.06348.x (PMC2610381; doi:10.1111/j.1365-2958.2008.06348.x)
Supplement: Supplementary file 1 [file mmi0069-1121-SD1.pdf]

Identification and characterisation of two trypanosome TFIIS proteins exhibiting particular domain architectures and differential nuclear localisations

Pierrick Uzureau[1,†], Jan-Peter Daniels[2,†], David Walgraffe[1], Bill Wickstead[2], Etienne Pays[1], Keith Gull[2,‡*], Luc Vanhamme[1,‡*].

[1]*Laboratoire de Parasitologie Moléculaire, ULB IBMM, rue des Pr Jeneer et Brachet 12, B-6041 Gosselies, Belgium.*

[2]*Sir William Dunn School of Pathology, University of Oxford, South Parks Road, OX1 3RE Oxford, UK*

† The two authors contributed equally to this work

‡ The two authors contributed equally to this work

*For correspondence. Email keith.gull@path.ox.ac.uk; Tel. (+44) 01865 285455; Fax (+44) 01865 285691; or luc.vanhamme@ulb.ac.be; Tel. (+32) 26 50 96 64; Fax (+32) 26 50 97 50

Supplemental Material

Suppl. Fig. 1. Characterisation of the TFIIS domains in TbTFIIS1 and TbTFIIS2-1. An alignment of the sequences in Fig. 1 is shown. The *T. cruzi* sequences were omitted because of high similarity to the *T. brucei* ones. Unaligned stretches were replaced with brackets indicating the number of amino acids trimmed. Domain I sequences appear to be better conserved across eukaryotes than domain II sequences.

Suppl. Fig. 2 . Identification of the TbTFIIS2-1 PWWP domain. A CLUSTAL W alignment was performed with the PWWP motif of the previously characterized *S. pombe* YH57 (SpYH57, accession number O94312), *M. musculus* HRP-3 (MmHRP-3, NP_038914.2) and DNM3B (MmDNM3B, O88509). Two *T. brucei* proteins with unknown function, namely TbTFIIS2-2 and Tb927.7.2520, were found to contain a PWWP domain and were added to the alignment. Identical and similar residues are shaded dark and gray, respectively. Amino acids N-terminal of the star have been truncated in the TY-YFP-TFIIS2-1(DPWWP) cell lines.

Suppl. Fig. 3. Comparison of the subnuclear localisations of TY-YFP-tagged TbTFIIS1 and TbTFIIS2-1 to the DAPI signal pattern. A. Procyclic cells labeled with BB2 (anti-TY) monoclonal antibody. B. Bloodstream-form cells processed in the same manner. On the merged images, FITC is pseudo-colored in green and DAPI in blue. Marks have been added to some images to aid in comparison. Both TFIIS proteins were found to have a tendency to be excluded from areas that were strongly stained with DAPI and might therefore correspond to heterochromatin. Bars, 1 µm.

Suppl. Fig. 4. Comparison of the subnuclear localisations TY-YFP-tagged full-length and ΔPWWP TbTFIIS2-1. A. Immunoblot analysis of whole-cell extracts from procyclic cells expressing TY-YFP-tagged full-length or ΔPWWP TFIIS2-1, respectively. Proteins were detected with monoclonal BB2 (anti-TY) antibody. B. Immunofluorescence on the same cell lines using BB2 (anti-TY) antibody. No differences between the subnuclear patterns of the two fusion proteins were detected. Bars, 1 µm.

Suppl. Fig. 5. Accuracy of the Celltiter cell proliferation assay for procyclic form trypanosomes. The measured optical density at 490 nm (OD_{490}) is plotted against the counted corresponding cell density of wild type trypanosomes. The square, diamond and triangle symbols correspond to three independent experiments.

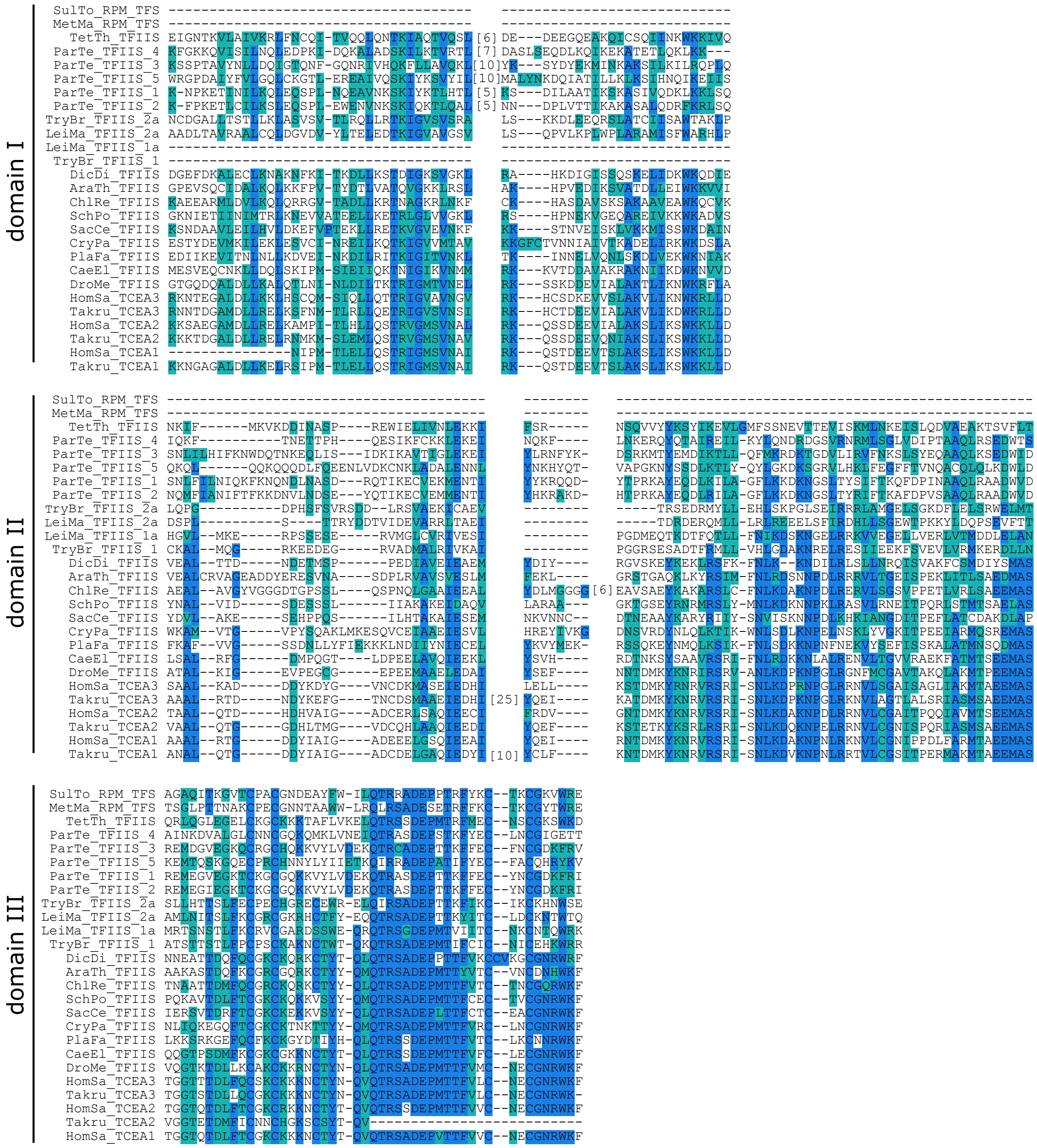

Suppl. Fig. 1

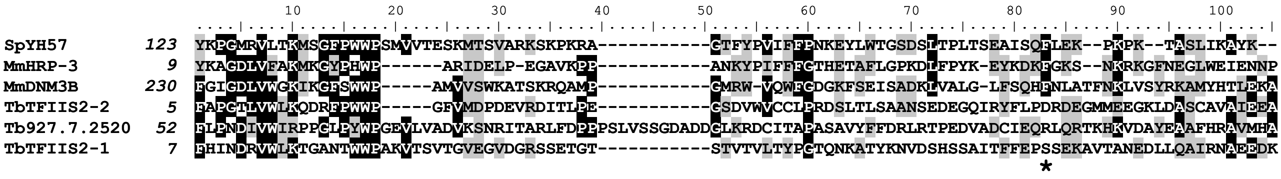

Suppl. Fig. 2

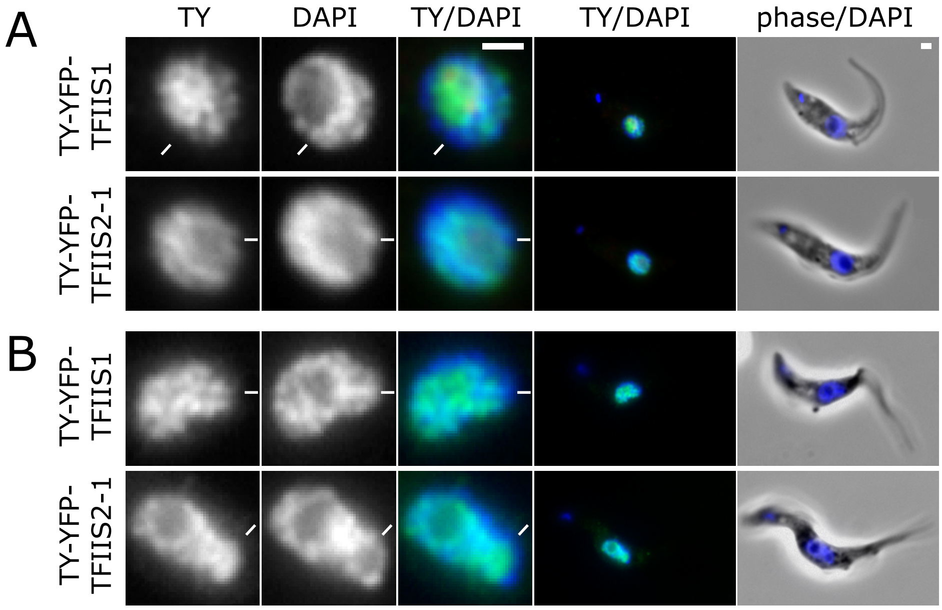
A
B
TY
DAPI
TY/DAPI
TY/DAPI
phase/DAPI
TY-YFP-TFIIS1
TY-YFP-TFIIS2-1
TY-YFP-TFIIS1
TY-YFP-TFIIS2-1

Suppl. Fig. 3

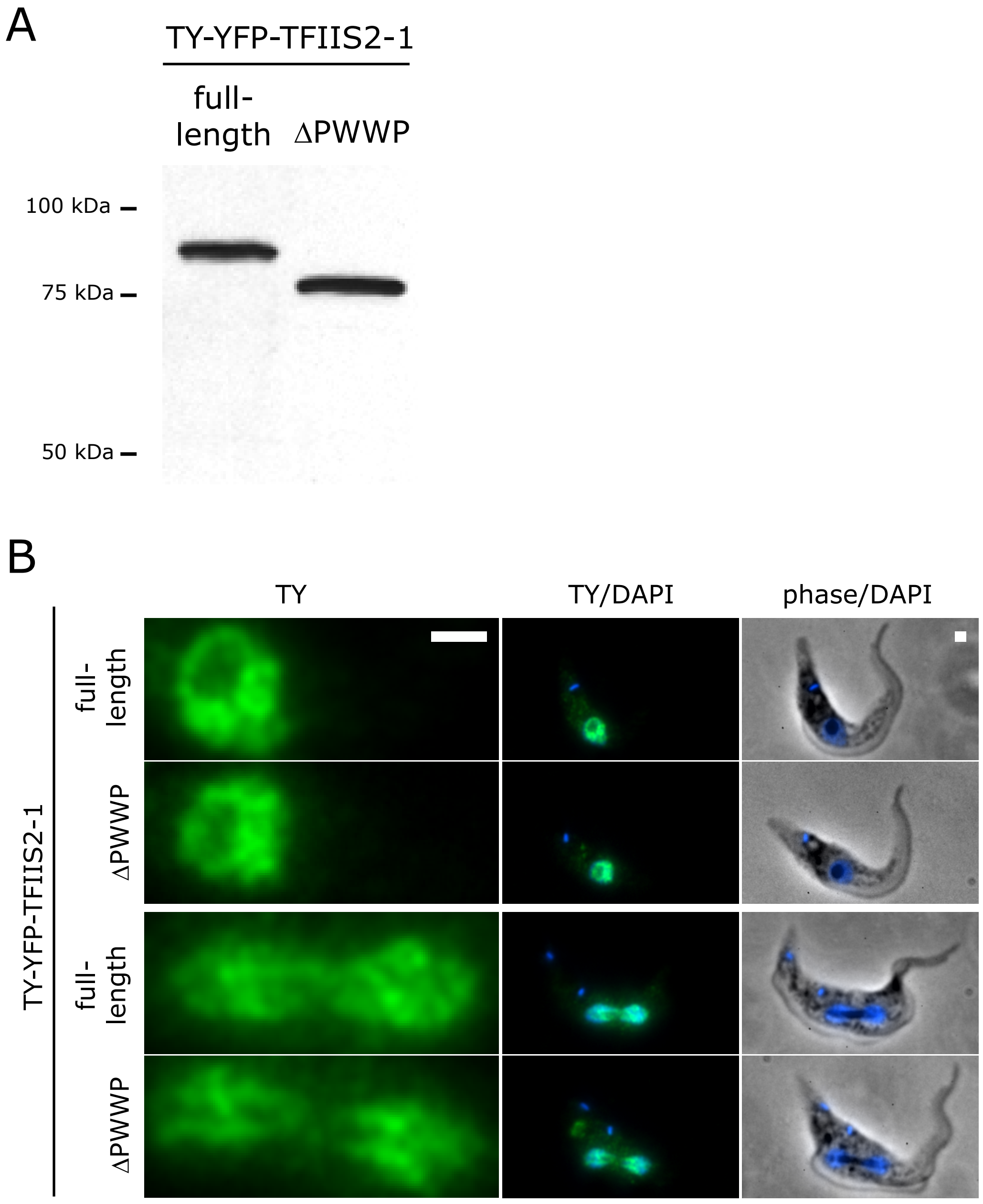
A
TY-YFP-TFIIS2-1
full-length
ΔPWWP
100 kDa
75 kDa
50 kDa
B
TY
TY/DAPI
phase/DAPI
TY-YFP-TFIIS2-1
full-length
ΔPWWP
full-length
ΔPWWP

Suppl. Fig. 4

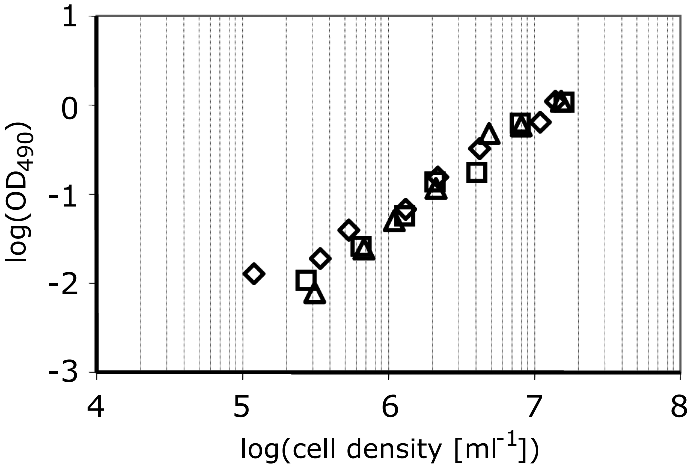

Suppl. Fig. 5

Supplemental Table 1. Sources, versions and web references of predicted proteomes used in this study.

Organism	Source[1]	Version	Web reference
Arabidopsis thaliana	The Arabidopsis Information Resource (TAIR)	TAIR6_pep_20051108	www.arabidopsis.org/
Caenorhabdatis elegans	WormBase	WS150	www.sanger.ac.uk/Projects/C_elegans/WORMBASE/
Chlamydomonas reinhardtii	Joint Genome Institute (JGI)	v3.0	genome.jgi-psf.org/Chlre3/Chlre3.home.html
Cryptosporidium parvum	cryptoDB	3.3	www.cryptodb.org/cryptodb/
Cyanidioschyzon merolae	C. merolae genome project	?	merolae.biol.s.u-tokyo.ac.jp/
Dictyostelium discoideum	dictyBase	primary	www.dictybase.org/
Drosophila melanogaster	Ensembl	BDGP4.2	www.ensemble.org/info/data/
Homo sapiens	Ensembl	NCBI 36	www.ensemble.org/info/data/
Leishmania major	geneDB	v5	www.genedb.org
Methanosarcina mazei	Goettingen Genomics Laboratory (G2L)	Goe1	www.g2l.bio.uni-goettingen.de/
Plasmodium falciparum	geneDB	3D7 v2.1.1	www.genedb.org
Paramecium tetraurelia	ParameciumDB	v1	paramecium.cgm.cnrs-gif.fr/
Saccharomyces cerevisiae	Ensembl	SGD1	www.ensemble.org/info/data/
Schizosaccharomyces pombe	geneDB	-	www.genedb.org
Sulfolobus tokodaii	Database Of the Genomes Analyzed at NITE (DOGAN)	-	www.bio.nite.go.jp/dogan/Top
Takifugu rubripes	Fugu Genome Project	v4	www.fugu-sg.org
Tetrahymena thermophila	Tetrahymena Genome Database (TGD)	Predictions_Aug_2004	www.ciliate.org/
Trypanosoma brucei	geneDB	v4	www.genedb.org
Trypanosoma cruzi	TcruziDB	v5.0	www.tcruzidb.org

[1] nb: Source of data does not necessarily correlate with sequencing centre(s). See individual projects for full acknowledgements of participants.

Supplemental Table 2. Accession numbers or genome IDs for dataset of protein sequences used for the phylogenetic analysis in this study.

**sequence name**	**accession number/genome ID**
**AraTh_TFIIS**	NP_181390
**CaeEl_TFIIS**	NP_495941
**ChlRe_TFIIS**	138155
**CryPa_TFIIS**	XP_627328
**DicDi_TFIIS**	XP_642233
**DroMe_CG3710-PA**	NP_476967
**DroMe_CG8117-PA**	NP_573049
**HomSa_TCEA1**	NP_006747
**HomSa_TCEA2**	NP_003186
**HomSa_TCEA3**	NP_003187
**LeiMa_TFIIS1_1**	CAJ04034
**LeiMa_TFIIS2_1**	CAJ06790
**MetMa_RPM_TFS**	NP_633422
**ParTe_TFIIS_1**	XP_001458090
**ParTe_TFIIS_2**	XP_001439670
**ParTe_TFIIS_3**	XP_001455726
**ParTe_TFIIS_4**	XP_001453275
**ParTe_TFIIS_5**	XP_001460845
**PlaFa_TFIIS**	XP_001349051
**SacCe_TFIIS**	NP_011472.1
**SchPo_TFIIS**	NP_593623
**SulTo_RPM_TFS**	NP_378260
**TakRu_TCEA1**	SINFRUP00000151201
**TakRu_TCEA2**	SINFRUP00000162345
**TakRu_TCEA3**	SINFRUP00000174721
**TetTh_TFIIS**	XP_001032085
**TryBr_TFIIS1**	XP_828571
**TryBr_TFIIS2_1**	XP_951597
**TryCr_TFIIS1_1**	XP_809248
**TryCr_TFIIS2_1**	XP_806892
